# Deep Sequencing-Based Analysis of the *Cymbidium ensifolium* Floral Transcriptome

**DOI:** 10.1371/journal.pone.0085480

**Published:** 2013-12-31

**Authors:** Xiaobai Li, Jie Luo, Tianlian Yan, Lin Xiang, Feng Jin, Dehui Qin, Chongbo Sun, Ming Xie

**Affiliations:** 1 Institute of Horticulture, Zhejiang Academy of Agricultural Sciences, Hangzhou, People’s Republic of China; 2 Department of Gastroenterology, The First Affiliated Hospital, College of Medicine, Zhejiang University, Hangzhou, People’s Republic of China; 3 College of Life Sciences, Hubei University, Wuhan, People's Republic of China; National Rice Research Center, United States of America

## Abstract

*Cymbidium ensifolium* is a Chinese *Cymbidium* with an elegant shape, beautiful appearance, and a fragrant aroma. *C. ensifolium* has a long history of cultivation in China and it has excellent commercial value as a potted plant and cut flower. The development of *C. ensifolium* genomic resources has been delayed because of its large genome size. Taking advantage of technical and cost improvement of RNA-Seq, we extracted total mRNA from flower buds and mature flowers and obtained a total of 9.52 Gb of filtered nucleotides comprising 98,819,349 filtered reads. The filtered reads were assembled into 101,423 isotigs, representing 51,696 genes. Of the 101,423 isotigs, 41,873 were putative homologs of annotated sequences in the public databases, of which 158 were associated with floral development and 119 were associated with flowering. The isotigs were categorized according to their putative functions. In total, 10,212 of the isotigs were assigned into 25 eukaryotic orthologous groups (KOGs), 41,690 into 58 gene ontology (GO) terms, and 9,830 into 126 Arabidopsis Kyoto Encyclopedia of Genes and Genomes (KEGG) pathways, and 9,539 isotigs into 123 rice pathways. Comparison of the isotigs with those of the two related orchid species *P. equestris* and *C. sinense* showed that 17,906 isotigs are unique to *C. ensifolium*. In addition, a total of 7,936 SSRs and 16,676 putative SNPs were identified. To our knowledge, this transcriptome database is the first major genomic resource for *C. ensifolium* and the most comprehensive transcriptomic resource for genus *Cymbidium*. These sequences provide valuable information for understanding the molecular mechanisms of floral development and flowering. Sequences predicted to be unique to *C. ensifolium* would provide more insights into *C. ensifolium* gene diversity. The numerous SNPs and SSRs identified in the present study will contribute to marker development for *C. ensifolium*.

## Introduction

*Cymbidium ensifolium*, is a diploid plant with an estimated haploid genome size of 4,000 Mb and 2n = 2x = 40 chromosomes [1,2]. *C. ensifolium* and similar oriental *Cymbidium* species, such as *C. sinense, C. faberi*, and *C. goeringii*, are under subgenus *Jensoa* of genus *Cymbidium* in the orchid family (Orchidaceae) [3]. *C. ensifolium*, found throughout Indochina, China, Japan, Borneo, New Guinea, and the Philippines, is one of the most widespread and diverse *Jensoa* species [4]. *C. ensifolium* cultivation in China dates back 2000 years. The flowers have an elegant posture, they range from 4 cm to 6 cm in length, and are borne well-spaced on an upright spike of three to eight or more [5]. *C. ensifolium* is easily propagated from new shoots and often blossoms more than twice throughout the year. *C. ensifolium* and many of its hybrids retain their flowers for long periods. These features make *C. ensifolium* an important primary parent, and contribute to the *Cymbidium* breeding pool for long-term studies, as demonstrated by elite cultivars such as *Cymbidium* Super Baby (× Babylon), *Cymbidium* Chocolate Soldier (× Volcano), and *Cymbidium* Tender Love (× *parishii*) in circulation [5]. Therefore, *C. ensifolium* takes a bigger share of the orchid flower market than other Chinese *Cymbidiums*.

Orchid flowers owe their unique appearance to their evolutionary success, and they are valued both for their beauty and their economic importance. Expression studies in other species indicate that the identity of floral organs is specified by the interaction of five different DEFICIENS-like MADS-box genes and exemplified by the ABCDE model of floral development [6-8]. For example, class A genes (e.g., *APETALA1*, *AP1*) control sepal development and they interact with class B genes (e.g., *PISTILLATA*, *PI*, and *APETALA3*, *AP3*) to regulate petals formation. Class B and class C genes (e.g., *AGAMOUS*, *AG*) interact to control stamen development. Class C genes determine carpel formation alone. Class D genes (e.g., *SEEDSTICK*, *STK* and *SHATTERPROOF*, *SHP*) specify the identity of the ovule within the carpel, and class E genes (e.g., *SEPALLATA*, *SEP*) are necessary for the proper formation and organization of all floral organs. The initiation of flowering in Arabidopsis is carried out through four genetic pathways: gibberellin, autonomous, vernalization, and light-dependent pathways [9,10]. These processes are integrated by the function of *Flowering Locus D (Fld*), *Flowering Locus T* (*FT*), *Flowering Locus E* (*FE*), *Flowering Wageningen* (*FWA*), *Protodermal Factor2* (*PDF2*), and Suppressor of Overexpression of Co1 (SOC1). The integrated signals of the floral induction are transmitted to the floral meristem identity genes *Leafy* (*LFY*) and *Apetala1* (*AP1*) genes, which are responsible for floral morphogenesis [10]. Determining homologues involved in *Cymbidium* flowering and flower development is the initial step for molecular-assisted selection (MAS).

Although much effort has been devoted to the cloning and identification of key genes involved in floral development and flowering of *Cymbidium* species [11-13], a comprehensive description of the genes expressed in *C. ensifolium* remains unavailable. The National Center for Biotechnology Information (NCBI) currently contains very limited *Cymbidium* sequence information, i.e., 692 nucleotide sequences and 78 expressed sequence tags (ESTs) (http://www.ncbi.nlm.nih.gov/nucest?term=cymbidium%5BOrganism%5D, verified 2013). *Phalaenopsis*, as well as *Cymbidium*, is a genus under the Epidendroideae subfamily of the orchid family. *Phalaenopsis equestris* has two bacterial artificial chromosome (BAC) libraries [14]. Numerous studies have developed EST resources for orchids using Sanger sequencing [15-18]. Approximately 12,000 ESTs, including 5,593 from *P. equestris*, 2,359 from *P. bellina*, 1,080 from *Oncidium* Gower Ramsey, and 2,132 from Vanda Mimi Palmer, have been deposited in public databases [19]. Recently, 206,960 ESTs were released from the pool containing *P. equestris*, *P. aphrodite* subsp. *formosana*, and *P. bellina* [19]. A total of 50,908 contig sequences were from *Oncidium* Gower Ramsey [20]. A total of 121,917 unique transcripts were identified for the *Ophrys* species, namely, *O. exaltata*, *O. garganica*, and *O. sphegodes*, using a combination of next-generation sequencing (454 and Solexa) and Sanger sequencing [21]. However, these species are genetically distant from *C. ensifolium* [22] and their sequence shares a relatively less similarity with *C. ensifolium*. Therefore, researchers urgently need a collection of EST sequences for *C. ensifolium* to facilitate whole genome annotation, molecular marker development, and studies on *C. ensifolium* floral traits*.*

Transcriptomes provide information regarding gene expression, gene regulation, and amino acid content of proteins at specific developmental stages or under certain physiologic conditions. Meanwhile, the data collections are also valuable for gene annotation and discovery [23,24], comparative genomics [25], development of molecular markers [26,27], and population genomics studies on genetic variation associated with adaptive traits [28]. Non-model organisms have to refer to the genome of the closest related species because of the lack of well-defined genomic references 29,30. However, a well-defined genome or a reference genome was unavailable for orchids until recently. Thus, researchers employ the assembly-first (de novo) method, which directly assembles transcripts using a high number of reads. In previous studies, many de novo assemblies for non-model organisms have been carried out based on Roche 454 pyrosequencing technology (currently about 500 bp) because it generates considerably longer reads than Illumina SOLEXA (approximately 100 bp) and ABI SOLiD technologies (approximately 50 bp). However, the high cost of reagents hinders the use of Roche 454 for complex uncharacterized genomes. Recently, software such as MIRA [31], Velvet [32], Oases [33], ABySS [34], Trans-ABySS [35], SOAPdenovo6 [36], and Trinity [37] have been developed specifically for RNA-Seq assembly using short sequence reads, and have been widely and successfully applied in various experiments [31-37]. Notably, Trinity has proven useful in non-model plant sequence assembly with Illumina reads from transcriptome data, with a sensitivity similar to methods that rely on genome alignments [37]. Therefore, Trinity helps overcome the disadvantages of short-read technologies to a certain degree.

In this report, we provide the *C. ensifolium* transcriptome from flower buds and mature flower, with 9.52 Gb of filtered nucleotides. The floral transcriptome was sufficiently comprehensive for gene discovery and analysis of major metabolic pathways associated with flower traits. As a resource, the transcriptome may be useful for genomic assembly, transcriptomic assembly, and microarray development in future studies. Based on transcriptome data, we identified a large number of genic-SSRs and genic-SNPs, which will increase the number of molecular markers, facilitate gene mapping, and genetic diversity analysis for *C. ensifolium.*

## Materials and Methods

### Plant material and RNA extraction

**Figure 1:**
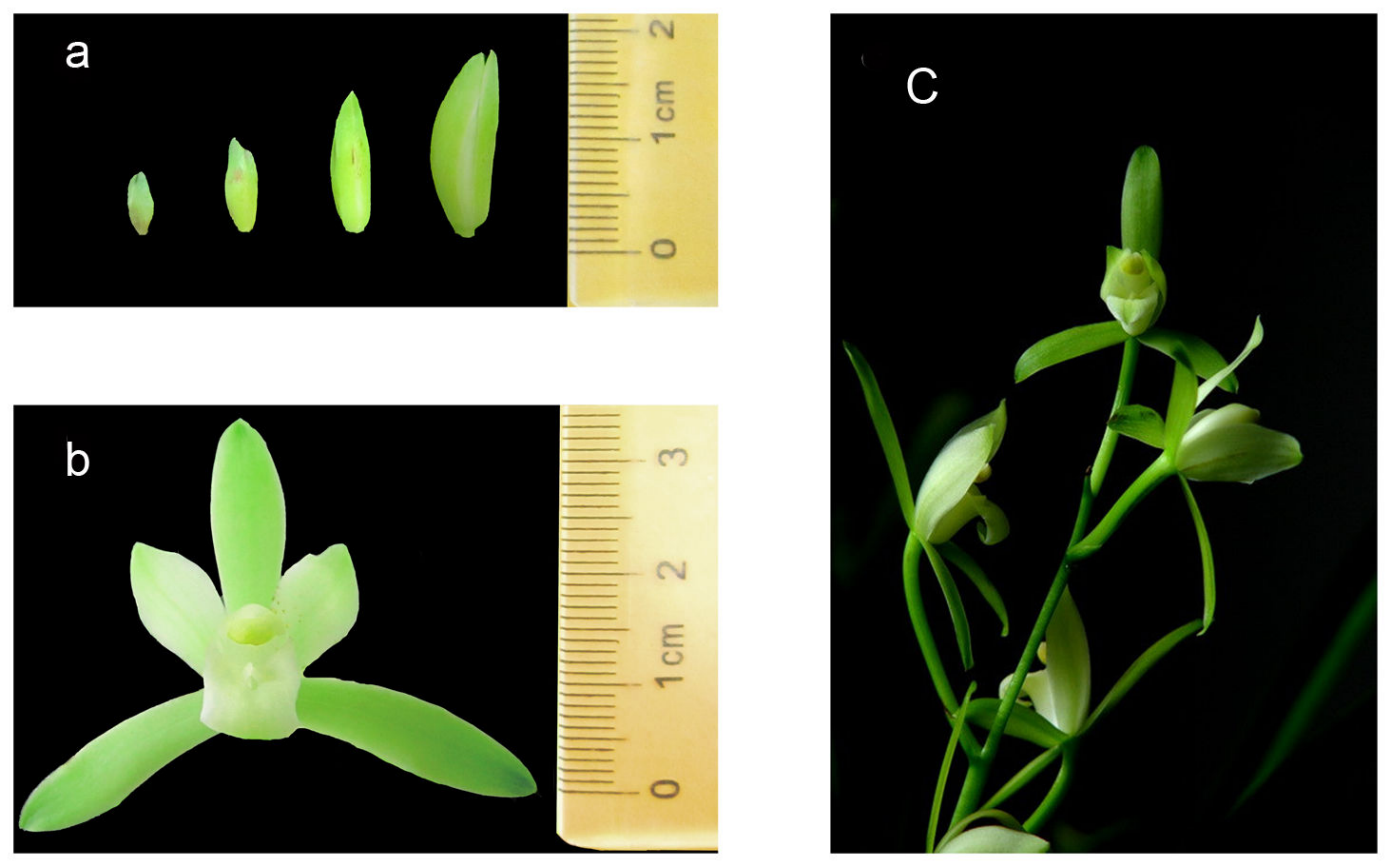
Four developmental stages of the flower bud and mature flower in *C. ensifolium* *a*: Stage 1 <0.5 cm; Stage 2, 0.5 cm to 1.0 cm; Stage 3, 1.0 cm to 2.0 cm; Stage 4, 2.0 cm to 3.0 cm; *b*: mature flowers; *c*: inflorescence.

Native cultivars of *C. ensifolium* “Tiegusu” with light green flowers were grown in a greenhouse at the Zhejiang Academy of Agricultural Sciences (Hangzhou, China) under natural light at 23 °C to 28 °C. “Tiegusu” is one of most widely known commercial cultivars in China. To cover as many transcripts as possible involved in floral development and flowering, tissues were collected from four stages of flower bud development (stage 1: <0.5 cm; stage 2: 0.5 cm to 1 cm; stage 3: 1 cm to 2 cm; stage 4: 2 cm to 3 cm) and from mature flowers (Figure 1). Each tissue sample consisted of a mixture of five plants. Total RNA was isolated from each sample using TRIzol® reagent (Invitrogen, CA, USA) and treated with RNase-free DNase I (TaKaRa) for 45 min according to the manufacturer’s protocol. The RNA obtained from four buds and mature flowers were subsequently pooled and used in cDNA library construction and Illumina deep sequencing.

### cDNA Library Construction and Sequencing

Illumina sequencing was performed at Shanghai Majorbio Bio-pharm Biotechnology Co., Ltd. (Shanghai, China) according to the manufacturer’sinstructions (Illumina, San Diego, CA) [38]. Firstly, mRNA containing poly-(A) tails was isolated from 20 μg of total RNA using Sera-mag magnetic oligo-(dT) beads (Illumina). To avoid priming bias, the purified mRNA was fragmented into small pieces (100 bp to 400 bp) using divalent cations at 94 °C for 5 min. Double-stranded cDNA was synthesized using a SuperScript double-stranded cDNA synthesis kit (Invitrogen, CA) with random hexamer primers (Illumina). The synthesized cDNA was subjected to end-repair and phosphorylation, and the repaired cDNA fragments were 3′-adenylated with Klenow exo- (3′ to 5′ exo minus, Illumina). Illumina paired-end adapters were ligated to the ends of the 3′-adenylated cDNA fragments. To select the proper templates for downstream enrichment, the ligation products were purified on 2% agarose gel. The cDNA fragments (approximately 200 bp) were excised from the gel. Fifteen rounds of PCR amplification were carried out to enrich the purified cDNA template using PCR primers PE 1.0 and 2.0 (Illumina) with fusion DNA polymerase. Finally, the cDNA library was constructed with 200 bp insertion fragments. After validation on an Agilent Technologies 2100 Bioanalyzer, the library was sequenced using Illumina HiSeq™ 2000 (Illumina Inc., San Diego, CA, USA). The following workflow was employed: template hybridization, isothermal amplification, linearization, blocking, sequencing primer hybridization, and sequencing on the sequencer to obtain the reads. After completion of the first read, the templates were regenerated in situ to enable a second read from the opposite end of the fragments. Once the original templates are cleaved and removed, the reverse strands undergo sequencing-by-synthesis. 

### Data filtering, de novo assembly, and annotation

We stringently filtered the raw sequencing reads before transcriptome assembly. Reads with more than 10% of bases with quality score Q<25, non-coding RNA (such as rRNA, tRNA, and miRNA), ambiguous sequences represented as “N,” and adaptor contaminants were removed. Furthermore, we discarded the reads that failed to pass the Illumina failed-chastity filter according to the relation “failed-chastity ≤ 1,” with a chastity threshold of 0.6 on the first 25 cycles. High-quality reads were assembled de novo using Trinity with an optimized k-mer length of 25 [37]. All de novo assembled isotigs (contig combinations representing full mRNAs) were compared with protein databases, including the non-redundant database (http://www.ncbi.nlm.nih.gov/), using BLASTX with a significance cut-off *E*-value of 1e^-5^. For the non-redundant annotations, the BLAST2GO V. 2.4.4 was also used to obtain the Gene Ontology (GO) annotations of unique transcripts [39]. Metabolic pathway analysis was performed based on the pathways of *Arabidopsis thaliana* and *Oryza sativa* in the Kyoto Encyclopedia of Genes and Genomes (KEGG) [40,41]. The unigene sequences were also aligned to the KOG (Eukaryotic Orthologous Groups) database to predict and classify possible functions [42].

We comparatively analyzed the *C. ensifolium* sequences against those of *P. equestris* (including 13,738 FL-cDNA and 9,393 isotigs) and *C. sinense* (http://orchidbase.itps.ncku.edu.tw/est/home2012.aspx) [43] with a relatively high stringency (E-value, 1e^-5^ in BLAST V.2.2.25). The *C. ensifolium* isotigs with significant matches were subjected to GO analysis for functional classification. 

### Mining of microsatellites and SNP

MSATCOMMANDER V. 0.8.2 [44] was used to analyze the microsatellite (SSR) distribution. The minimum number of repeats for SSR detection were as follows: six for di-SSRs; and four repeats for tri-, tetra-, penta-, and hexa-SSRs. The open reading frame (ORF) and untranslated region (UTR) within the isotig were identified using Trinity [37]. The location of SSRs was estimated based on ORFs and UTRs. SSR-containing isotigs were annotated based on BLAST similarity searches described above. Primers for genic-SSRs in microsatellite sequences were designed with Primer3 [45], based on the following core criteria: a G/C content between 40% and 70%, an annealing temperature between 54°C and 63°C, a minimum product length of 100 bp, and a primer length of 18–24 nucleotides.

SNPs were detected based on alignment using BWA V. 0.5.9 [46] and SAMtools V. 0.1.18 [47]. From the ‘pileup’ output of SAMtools, VarScan V.2.2.7 filtered SNPs based on the following criteria: (1) the total coverage and the number of reads to cover a candidate SNP (>8 reads); (2) the base quality where base calls with low Phred quality (<25) were removed from the coverage; and (3) frequency of mutated bases higher than 30% among all reads covering the position.

## Results and Discussion

### Illumina paired-end sequencing and de novo assembly

**Figure 2:**
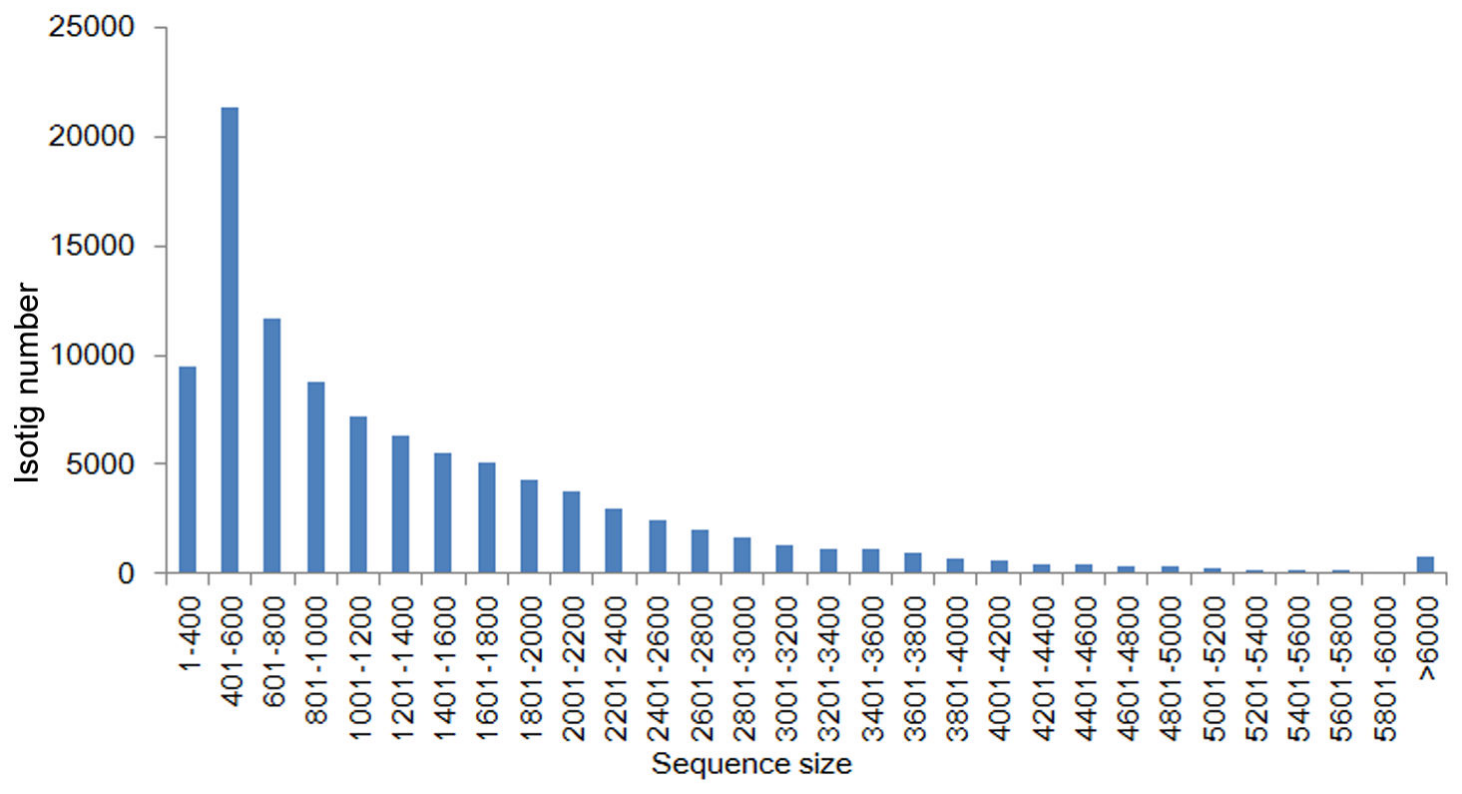
Isotig length distribution. The x-axis represents the sequence length in base pairs. The y-axis represents the number of isotigs relative to the sequence length.

Sequenced sample yielded 2 × 100 bp independent reads from either end of a cDNA fragment. After removal of adaptor sequences, ambiguous reads, and low-quality reads (Q-value < 25), we obtained a total of 98,819,349 of high-quality filtered reads containing 9,523,132,764 nucleotides (9.52 Gb), which were used for the subsequent assembly. An overview of the sequencing and assembly is summarized in Table 1 and Figure 2. All high-quality reads were assembled into 101,423 isotigs, with 139,385,689 total residues. The average isotig length was 1,374 bp and ranged from 351 bp to 17,260 bp. The 101,423 isotigs were derived from 51,696 genes, mostly from alleles or alternative splicing. The data were uploaded to the Website (http://orchidbase.itps.ncku.edu.tw/est/home2012.aspx) for public use (Accession: SRA098864).

**Table 1:** Summary of the sequence assembly before/after Illumina sequencing.

	Type	All numbers	Sequences (bp)	Mean length (bp)
Before assembly	Filtered reads	98,819,349	9,523,132,764	96
After assembly	Total genes:	51,696	-	-
	Total isotigs:	101,423	139,385,689	1,374
	Largest isotig:	-	17,260	-
	Smallest isotig:	-	351	-

### Functional annotation of *Cymbidium ensifolium* flower transcriptome

The isotig annotations provide functional information regarding the *C. ensifolium* transcriptome, such as KOG clusters, GO, and KEGG pathway information. We predicted protein functions using the annotation of the most similar proteins. Distinct gene sequences were first searched using BLASTX against the Nr database. The results show that 41,873 isotigs (41.3% of all isotigs) had hits that exceeded the E-value threshold.

**Figure 3:**
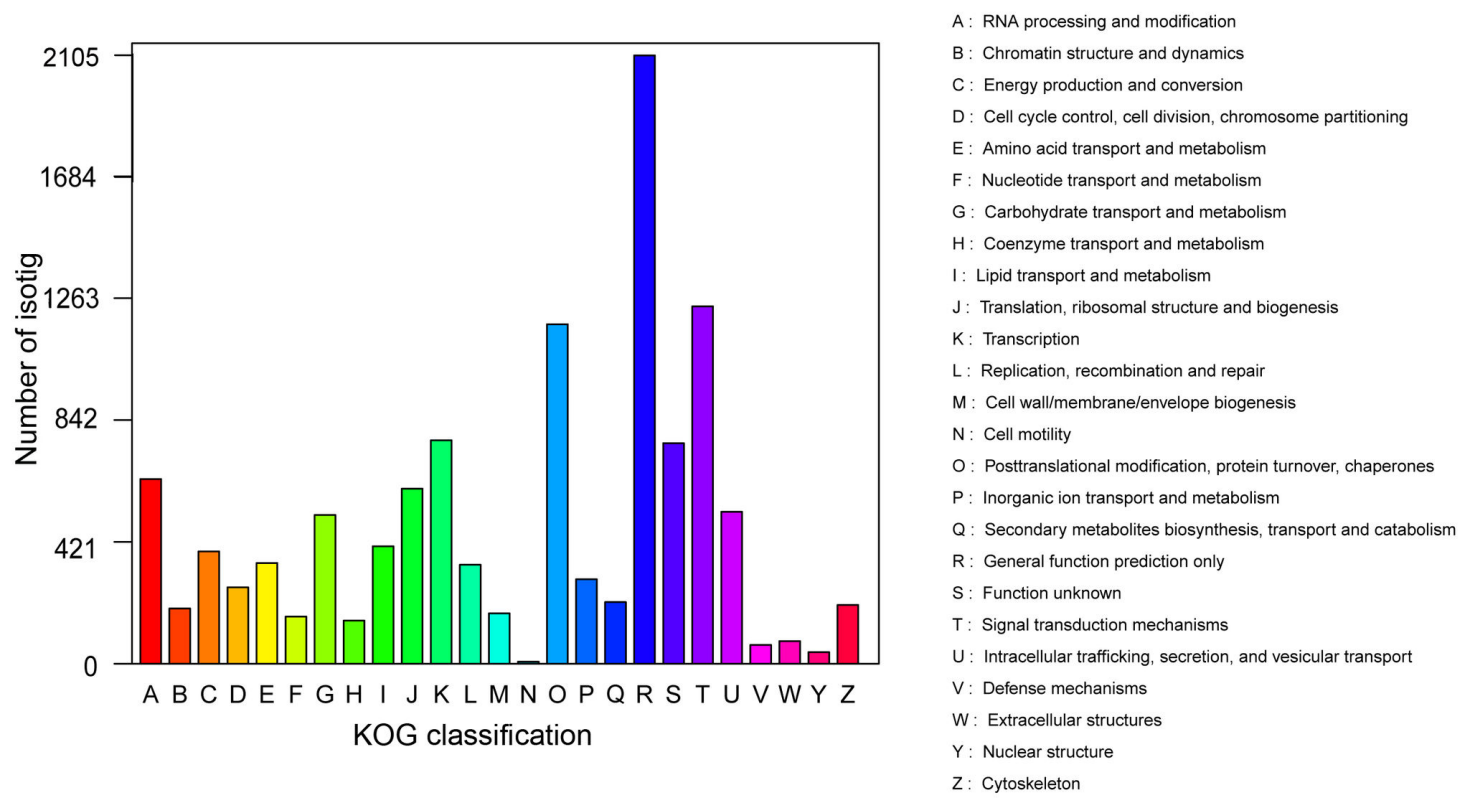
Histogram of KOG classification. All isotigs were aligned with genes in the KOG database to predict and classify possible functions. Of the 41,873 isotigs with nr hits 10,212 were assigned to 25 KOG classifications.

The KOG is a major update of the previously developed system for delineating Clusters of Orthologous Groups of proteins (COGs) from the sequenced genomes of prokaryotes and unicellular eukaryotes and the construction of clusters of predicted orthologs for seven eukaryotic genomes [48]. The KOG database, similar to COG, provides information on classifications of orthologous gene products, including the genome-wide coding proteins and the evolutionary relationships [42,48]. The classification is based on the assumption that every protein evolved from an ancestor protein. *C. ensifolium* isotigs were searched against the KOG database to predict and classify their possible functions. Out of 41,873 hits in the Nr databases, 10,212 sequences were classified into 25 KOG categories (Figure 3). Among the 25 KOG categories, “general function prediction only” was the largest group (2,102 isotigs; 20.58% of all isotigs), followed by “Signal transduction mechanisms” (1,235; 12.09%), “Post-translational modification, protein turnover, chaperones” (1,173; 11.48%). “Cell motility” was the smallest group (7; 0.07%), followed by “Chromatin structure and dynamics” (40; 0.39%) and “Extracellular structures” (78; 0.76%) (Figure 3).

**Figure 4:**
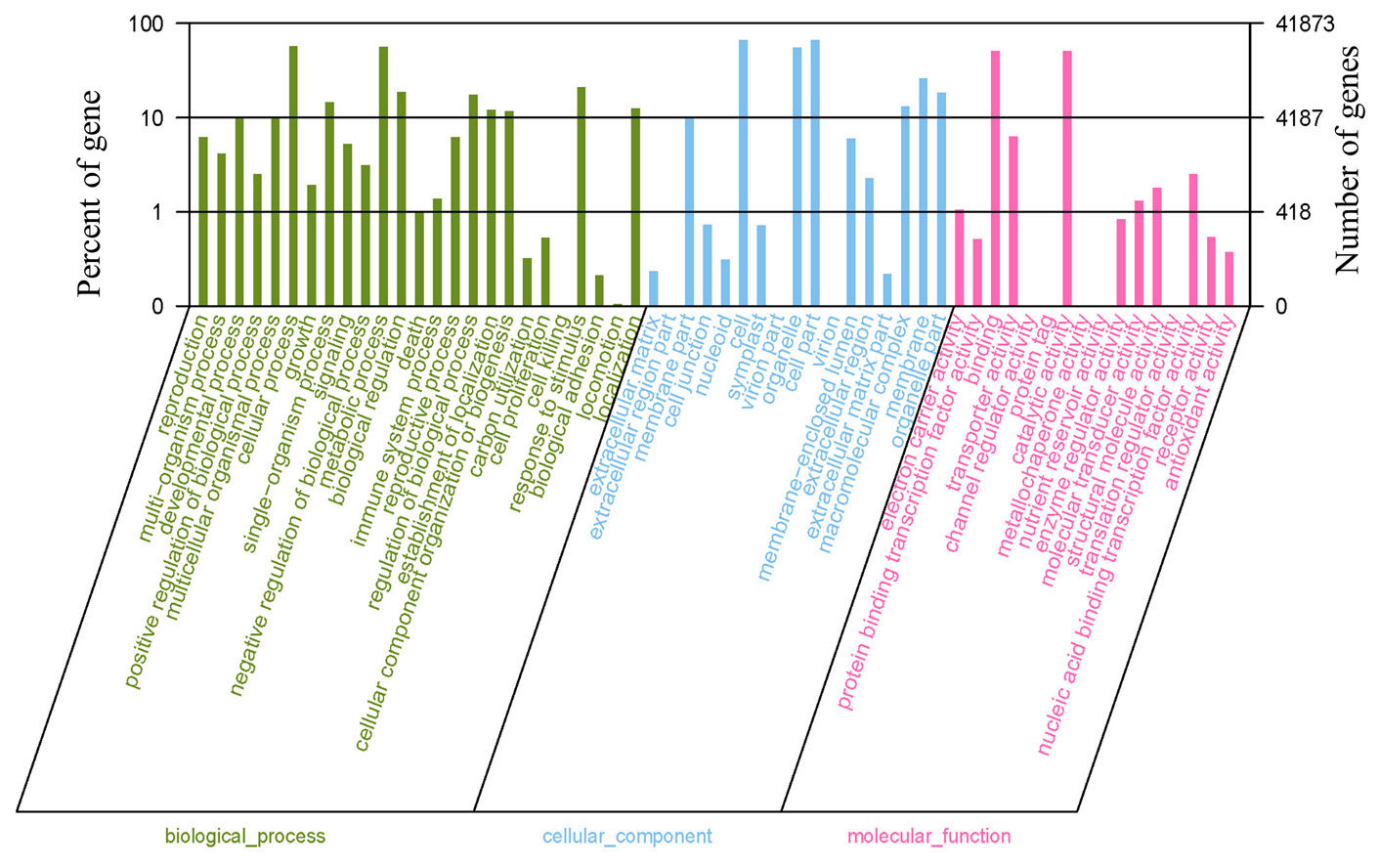
Gene Ontology classifications of assembled isotigs. A total of 41,873 isotigs with significant similarity to nr protein databases were assigned to gene ontology classifications.

GO is an internationally standardized gene function classification system that provides a comprehensive description of gene properties across species and databases. In the GO database, the genes are classified into three ontologies, i.e., molecular function, cellular components, and biological processes. The ontology consists of the basic units, i.e., GO terms or functional groups. In the present study, 41,690 isotigs were categorized into 58 GO terms in three GO ontologies (Figure 4). For molecular function, “catalytic activity” had the most number of isotigs (21,428), followed by “binding” (21,169). For cellular components, “cell” and “cell part” had the highest number of isotigs (both 27,687). For biological processes, “cellular process” had the highest number of isotigs (23,987), followed by “metabolic process” (23,481). 

The KEGG pathway database contains information on networks of intracellular molecular interactions, and their organism-specific variations [49]. To identify the biological pathways in *C. ensifolium*, we mapped the annotated sequences to the reference canonical pathways of model plants, such as Arabidopsis and rice. Referring to Arabidopsis pathways, 9,830 isotigs were found to be involved in 126 pathways (Table S1). The most highly represented was “metabolic pathways,” with 1,677 members, followed by “biosynthesis of secondary metabolites,” with 908 members. For the rice pathways, 9,539 isotigs were involved in 123 pathways (Table S2). Similarly, “metabolic pathways” was also the most represented pathway (1,378), followed by “biosynthesis of secondary metabolites” (714).

The isotigs with homeostatic functions comprised the majority of annotated genes, such as genes related to signal transduction mechanisms in the KOG analysis, genes with catalytic activity in the GO analysis, and genes involved in metabolic pathways and the biosynthesis of secondary metabolites. The annotation information could help in determining gene functions and metabolic pathways in *C. ensifolium*. 

### Functional genes involved in floral development and flowering

**Figure 5:**
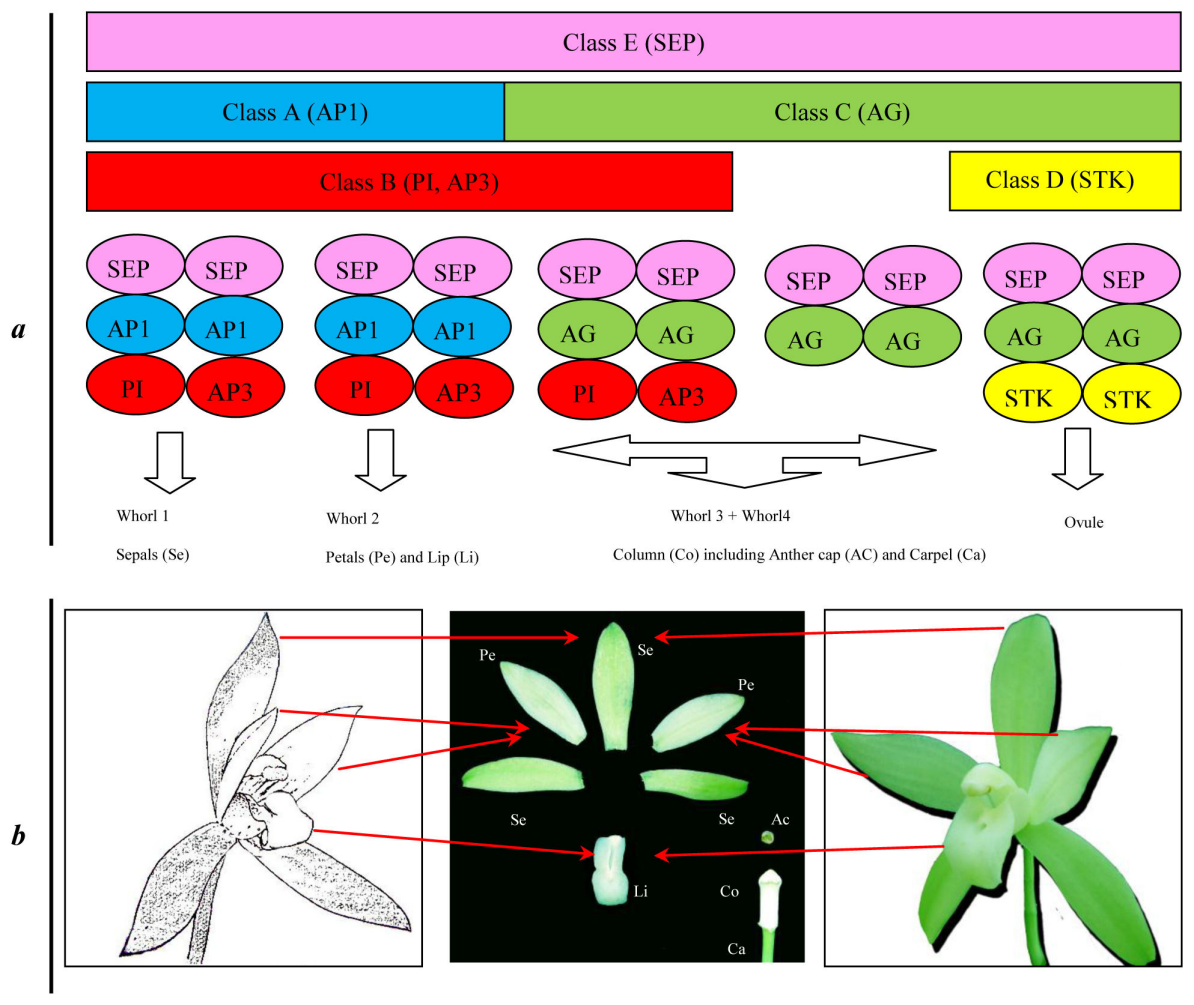
Diagram of the expanded ABCDE model of floral development. *a*. In the model, A, B, C, D, and E class MADS-box proteins interact, leading to the formation of homodimers and heterodimers called “floral quartets.” The complexes then activate floral organ-specific expression programs [6-8]. Class A genes (*APETALA1*, AP1) control sepal development and, class A and class B genes (e.g., *PISTILLATA*, *PI*, and *APETALA3*, AP3) jointly regulate petal formation. Class B and class C genes (e.g., AGAMOUS, *AG*) jointly mediate stamen development. Class C genes determine the formation of carpel alone. Class D genes (e.g., *SEEDSTICK*, *STK* and *SHATTERPROOF*, *SHP*) specify the identity of the ovule within the carpel. Class E genes (e.g., *SEPALLATA*, *SEP*) are necessary for the proper formation of all floral organs. In orchids, male and female tissues fuse into a gynostemium or column. The orchid code theory suggests that class B *AP3/DEF*-like genes play a crucial role in lateral petal and lip identity and the class PI/GLO-like genes and the A, C, D and E class genes have unchanged function [8,50-52]. *b*. Floral organs of *C. ensifolium*. Se: Sepals (whorl 1); Pe: Petals and Li: Lip (whorl 2); Co: Column including Ac: Anther cap and Ca: Carpel (whorl 3+Whorl4).

*Cymbidium*, like most orchids, has unique flowers consisting of three whorls of perianth (outer tepals or sepals, lateral inner tepals or lateral petals, a lip or labellum, and column or gynostemium). Previous studies suggested that the identity of flower organs is specified by the interaction of A, B, C, D, and E class *DEFICIENS*-like MADS-box genes [6-8]. In orchids, the class B gene AP3/DEF-like determines the identity of the lateral petals and lip, whereas the class B gene PI/GLO-like, with class A, C, D, and E genes, retain the function unchanged; they constitute the “Orchid code” (Figure 5) [8,50-52]. In the present study, we identified 158 putative MADS genes, including five classes of A (e.g. *AP1*) (44 isotigs), B (*AP3*, *PI/GLO*) (6), C (*AG*) (6), D (*STK*) (1), E (*SEP*, *AGL*) (31), and others (65) (Table S3). Some genes exhibited the greatest homology to genes in orchid family, such as *Cymbidium* (23 isotigs), *Dendrobium* (13), *Phalaenopsis* (8), *Agave* (5), *Aranda* (2), and *Gongora* (2). Others genes were more homologous to *Brachypodium distachyon* (15), *Eschscholzia californica* (16), *Triticum aestivum* (19), *Vitis vinifera* (21), *Zea mays* (12), and *Oryza sativa* (6). Further investigation on putative homologs of wheat, maize, or rice genes should provide interesting clues to floral development in *C. ensifolium*.

Approximately 118 isotigs were homologous to genes related to the flowering pathway (Table S4), e.g., *Flowering Locus D (FLD*) with one copy, *Flowering Locus T* (*FT*) with three copies, *Flowering Time Control* (*FCA*) with three copies, Forever Young Flower (FYF) with six copies, *Dicer-Like 3a* (*DCL3A*) with one copy, and *Vernalization Independent Insensitive 3* (*VIN3*) with eight copies. In Arabidopsis, *FT* and *FLD* are repressed by *FLC*, which is the major flowering repressor in the vernalization pathway [53]. *VIN3* is expressed specifically during a vernalizing cold treatment, and expression is completely abolished when plants are returned to a warm temperature [54]. The cold induction and transient nature of *VIN3* expression indicates that *VIN3* may be a part of the trigger to set in motion the molecular events that stably repress *FLC* during vernalization [53]. In *Cymbidium*, the *FT* homolog is suggested to be involved in the transition from the vegetative to reproductive phase [55]. *FYF* causes a significant delay in senescence and a deficiency of abscission in flowers of transgenic Arabidopsis [56].

Certain flowering genes are involved in organ development. A number of these genes are expressed in developing *C. ensifolium* flowers. For example, CO (CONSTANS) gene, a key regulator of flower photoperiodic responses in Arabidopsis, is expressed in the shoot apical meristems and leaves, as well as in inflorescences and roots [57]. In *Gossypium hirsutum*, a *CO* homolog is strongly expressed in flower buds and mature flowers, and weakly expressed in ovules [58]. *FCA* for flowering time control is expressed in the shoot apex, as well as in mature leaves, inflorescence, and roots [59]. In the present study, 27 putative CO-like homologs were identified, 6 of which were homologous to genes in the orchid family. The other 21 CO-like isotigs were homologous to genes in *Glycine max* (8 isotigs), *Arabidopsis thaliana* (7), *Brachypodium distachyon* (2), *Solanum lycopersicum* (1), *Vitis vinifera* (1), *Zea mays* (1) and *Oryza sativa* (1). Three *FCA* isotigs were homologous to those in *Vitis vinifera*.

The importance of GA in flower formation has also been demonstrated by inducing flowering during the normal vegetative growth stages of plants [60]. Our study identified 36 putative homologs of gibberellin (GA)-signaling pathway genes, including the gibberellin response modulators *Dwarf 8* (*D8*) (two copies), *GA3ox* (two copies), and *GAMYB* (two copies). In maize, the *D8* gene has been identified as an ortholog of the gibberellic acid insensitive (GAI) gene, a negative regulator of GA response in Arabidopsis [61]. In tobacco and rice, transcripts of *GA3ox* genes are localized in the pollen and tapetum of developing anthers [62,63], which indicates their involvement in stamen development. The MYB transcription factors contain specific DNA-binding domains and function as floral developmental regulators [64,65]. In Arabidopsis, *MYB33* and *MYB65* are both expressed at the shoot apex, as well as the petioles, wherein they induce elongation and erect growth. *MYB33* and *MYB65* are functionally redundant, and a double mutant causes defective anther development. *MYB33* may mediate GA signaling during flowering by binding to the promoter of the floral meristem-identity gene, *LEAFY* [66]. In addition, *MYB21*, *MYB24*, and *MYB57* are all DELLA-repressible GA-response genes that mediate stamen filament growth, and mediate stamen maturation through jasmonate [67].

### Comparative analysis with *P. equestris* and *C. sinense*

**Figure 6:**
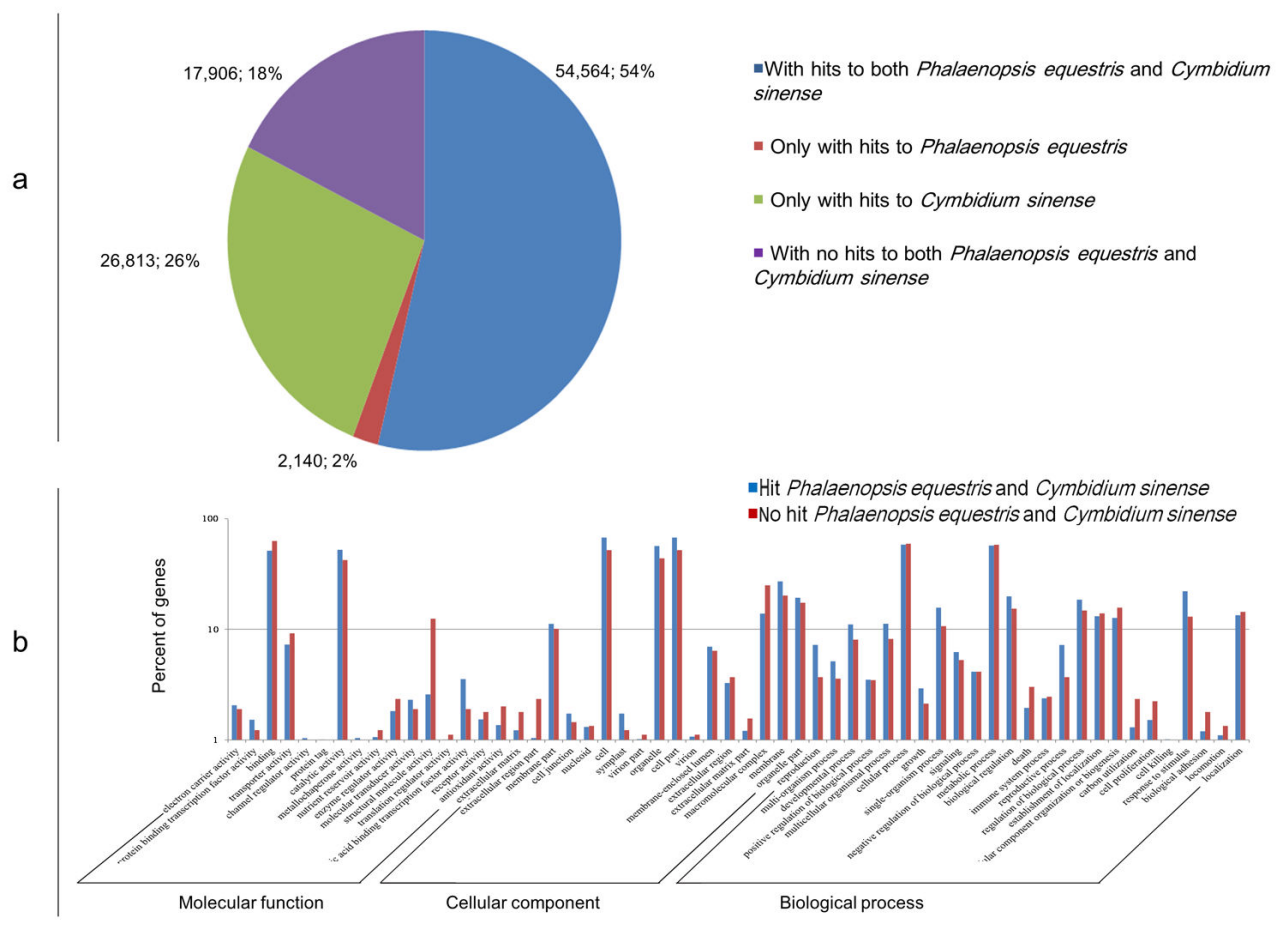
Comparison of *C. ensifolium* isotig similarity with *P. equestris* and *C. sinense* and GO functional classification. *a*. Similarity search of *C. ensifolium* sequences against *P. equestris* and *C. sinense* sequences. *b*. Functional classification of *C. ensifolium* isotigs with and without homologs with *P. equestris* and *C. sinense*.

*P. equestris* is a well-studied orchid species that has a large amount of available annotated sequencing data. In the present study, we compared the *C. ensifolium* transcriptome with the P. *equestris* transcriptome. The search results showed that 56,704 (55.91%) *C. ensifolium* isotigs have similarity hits with *P. equestris*. *C. sinense* as well as *C. ensifolium* is a members of the same genus; thus we compared their transcriptome datasets. The comparison indicated that 81,377 (80.24%) *C. ensifolium* isotigs significantly matched those of *C. sinense*. However, the *C. sinense* transcriptome consisted of 35,191,817 bp, which is significantly lower than the 139,385,689 bp of the *C. ensifolium* transcriptome. The average length of the *C. sinense* isotig was 345 bp, which is significantly shorter than the 1,374 bp in *C. ensifolium*. These isotigs had 0.31% ambiguous nucleotides represented by “N.” Among the aligned sequences, 54,564 (53.80%) isotigs had similarities with both *P. equestris* and *C. sinense*, whereas 17,906 (17.65%) isotigs had no similarity with either *P. equestris* or *C. sinense* (Figure 6A). These results indicate that the P. *equestris* and *C. sinense* dataset is still incomplete or that the unmatched *C. ensifolium* isotigs may be unique.

Based on the similarity search above, we conducted a GO analysis to compare the functional classification between the two groups of orchid isotigs, one including shared homologs with both *P. equestris* and *C. sinense*, and the other presumably including those unique to *C. ensifolium* (Figure 6B). The detailed results are listed in Table S5. Among the shared homologs, 40,982 of the 54,564 isotigs were assigned to one or more GO terms. The genes involved in metabolic processes and cellular processes were highly represented. For molecular functions, “catalytic activity” was the most prevalent GO term, followed by “binding.” For cellular components, the most represented category was “cells,” followed by “cell part” and “organelle” (Figure 6B). In the “unique group,” only 891 of the 17,906 isotigs were annotated through GO analysis, displaying a similar trend to the annotated shared homologs. Such a low annotation percentage may be caused by the relatively small amount of high-quality sequenced *C. ensifolium* genes deposited and annotated in public databases. Furthermore, a part of *C. ensifolium* genes share low sequence similarity with their homologs in well-studied model species, such as rice and Arabidopsis. The unique isotigs may be important for traits specific to *C. ensifolium*, such as special flower formation and scent production. Most predicted unique isotigs have not been characterized, but they represent a valuable resource for exploring the genetic diversity of *C. ensifolium* and for comparative genomic studies among orchids.

### Genic-SSR and genic-SNP identification

Genic markers are based on particularly expressed sequences; thus, they are potentially tightly linked with functional genes that may control certain important phenotypic characteristics [68]. In rice, variations in the number of GA or CT repeats in the 5′UTR of the waxy gene is correlated with amylose content [69]. Similarly, the microsatellite markers (CCG)n in the 5′UTRs of some ribosomal protein genes of maize are suggested to regulate fertilization [70]. In bread wheat, some genic-SSRs linked to gliadin or glutenin are associated with bread quality, and other markers are linked to stress responsive genes [71]. In Chinese winter wheat, 17 genic-SSRs are associated with seven yield traits [72]. The lack of cDNA data for *C. ensifolium* seriously hinders the development of *C. ensifolium* genic-SSRs. A total of 7,936 SSRs were identified in the present study, with one SSR locus for every 17.56 kb (kb/SSR). The average distance exceeds 0.92 kb/SSR to 1.72 kb/SSR in *Cryptomeria* EST library [73], 4.08 kb/SSR [74], and 7.04 kb/SSR [75] in the sesame transcriptome, 6.22 kb/SSR in the peanut transcriptome [76], 6.69 kb/SSR in *Epimedium* transcriptome [77]. The SSRs were distributed over 8,080 (7.97%) of the isotigs. The frequency was higher than 5.4% in *Pinus*, 7.0% in spruce, 4.5% in the *Cryptomeria* EST library [73], and 3.67% in the *Epimedium* transcriptome [77]. However, the frequency is lower than 8.93% in the sesame transcriptome [75], 8.45% in the peanut transcriptome [76], 13% [78] and 14% in the citrus transcriptome [79]. Although *C. ensifolium* SSR resources are as not as extensive as in many other species, the number of SSRs is still sufficiently large for identifying markers. 

Theoretically, the frequency of nucleotide repeats generally decreases with increasing length, i.e., dinucleotides > trinucleotides > tetranucleotides > pentanucleotides > hexanucleotide [77]. However, trinucleotide repeats are the most dominant SSRs in *C. ensifolium*, followed by dinucleotide, tetranucleotide, hexanucleotide, and pentanucleotide repeat units. These SSRs include 3,640 di-SSRs (45.87% of the total SSRs), 3,911 tri-SSRs (49.28%), 302 tetra-SSRs (3.81%), 44 pent-SSRs (0.55%), and 39 hexa-SSRs (0.49%) (Table 2). This trend is consistent with the results reported for other plant species [78,80-82]. The most abundant dinucleotide and trinucleotide motifs were AG/CT (69.81% in di-SSRs) and AAG/TTC (24.37% in tri-SSRs), respectively, which are consistent with previous reports [38,83-85]. 

**Table 2:** Summary of SSRs within the *C. ensifolium* transcriptome.

Type	5′UTR^a^	3′UTR^b^	CDS^c^	Undetermined^d^	Total
Dinucleotide	1,055	829	579	1,177	3,640
AC(GT)	39	56	13	166	274
AG(CT)	919	408	541	673	2,541
AT(AT)	93	363	24	334	814
CG(CG)	4	2	1	4	11
Trinucleotide	464	642	1,740	1,065	3,911
AAC(GTT)	54	77	46	334	511
AAG(CTT)	165	148	468	172	953
AAT(ATT)	63	280	33	290	666
ACC(GGT)	8	16	97	24	145
ACG(CGT)	10	1	34	4	49
ACT(AGT)	4	7	6	15	32
AGC(GCT)	26	19	179	25	249
AGG(CCT)	38	12	286	34	370
ATC(GAT)	51	72	267	134	524
CCG(CGG)	45	10	324	33	412
Tetranucleotide	46	93	14	149	302
Pentanucleotide	5	21	3	15	44
Hexanucleotide	5	4	24	6	39
total	1,575	1,589	2,360	2,412	7,936

^a^ 5′ or 3′ UTR : 5′ or 3′ untranslated region

^b^ CDS: the coding region of a gene

^c^ Undetermined: the corresponding SSRs extending over both coding and UTR regions

^d^ Sequence in bracket: the reverse-complement

**Figure 7:**
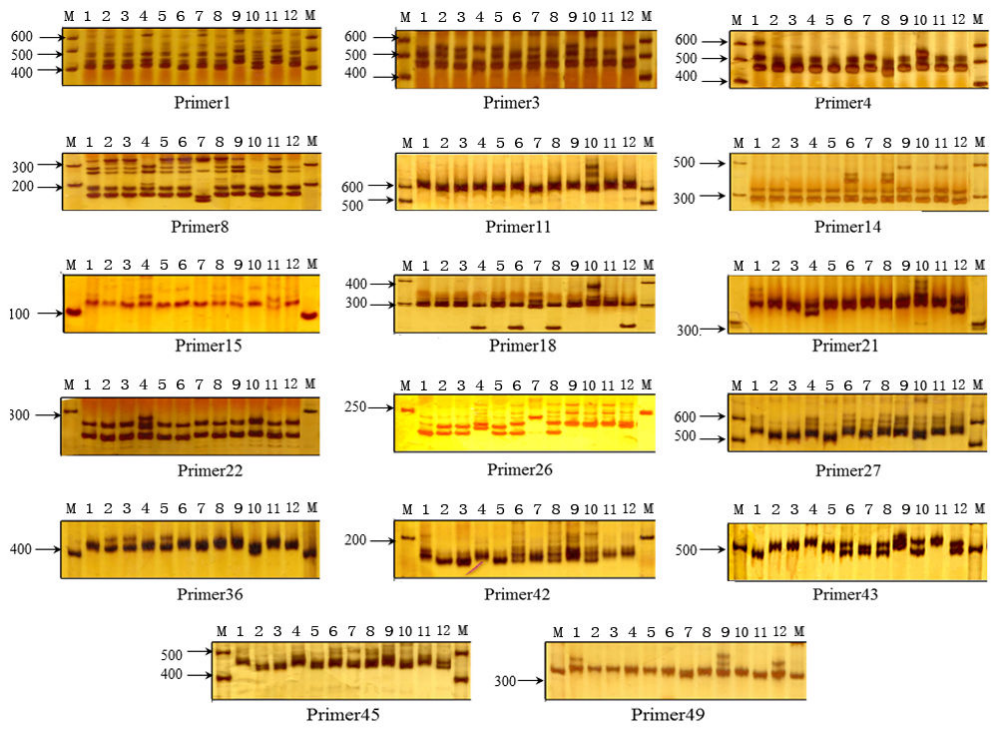
Polymorphism of the 17 genic-SSR among 12 *C. ensifolium* accessions. M: DNA marker; Lanes 1 ~ 12: Samples (Table S6).

The location of microsatellites is important for SSR marker development [86,87]. The estimated locations (coding, 5′UTR or 3′UTR) were obtained for 5,524 of the total 7,936 SSRs. Sequence information could not be determined for the remaining 2,412 SSR regions because the locations were extended over both estimated coding and non-coding regions. Most tri-SSRs (1,106 UTR-SSR vs. 1,740 coding-SSR) and hexa-SSRs (9 UTR-SSR vs. 24 coding-SSR) occurred more frequently in the coding regions. By contrast, the di-SSRs (1,884 UTR-SSR and 579 coding-SSR), tetra-SSRs (139 UTR-SSR and 14 coding-SSR), and penta-SSRs (26 UTR-SSR vs. 3 coding-SSR) were mostly distributed in the UTR rather than coding regions (Table 2). The observed phenomenon may be due to selective pressures on SSRs in the coding regions. The addition or deletion of trinucleotide repeats, i.e., tri-SSRs and hexa-SSR in the coding regions would not cause a frame shift mutation and, in most cases, have less detrimental effects on the gene product [73]. AT-rich motifs were abundant within 3′UTRs, e.g., 75.63% of AT and 74.47% of AAT were in 3′UTRs. The AT-rich motif within 3′UTRs are highly over-represented because of certain cis-acting elements, such as poly-A signal AAUAAA, which are involved in controlling mRNA stability [88]. The most common tri-SSRs in the coding region were AAG, which accounts for 59.92% of the total tri-SSRs. The dominant motif AAG in coding regions may be a result of the high frequency of its usage in translation, which was also found in Arabidopsis [89]. Trinucleotide types within coding regions and binucleotide types within non-coding regions should be prioritized for SSR marker development because they are potentially more polymorphic than other regions [87]. Here, we randomly chose 35 and 15 SSR within UTR and within CDS regions, respectively, which were subject to primer design and PCR amplification. Of 50 primer pairs, 46 produced clear bands and 17 were polymorphic (Figure 7; Table S7) among 12 *C. ensifolium* accessions (Table S6). The PCR success rate (92%) was in line with previously reported ratios of 60–92.2% amplification [71,74,90,91]. Therefore, the 7,936 potential genic-SSRs identified in this research will provide a wealth of resource for developing SSRs in *C.ensifolium*.

Similar to genic-SSRs, genic-SNPs are also useful for identifying genome colocation events between candidate genes or QTLs [92]. In apples, genic-SNPs have been linked to several genes that confer resistance to the apple scab fungal disease [93]. Other genes, such as ACC synthase (MDU73816, LG 15) [94] and allergen proteins (EB133053 and LG 13) [95], have been remapped using genic-SNPs. In Chinese cabbage, three SNPs were located in *BrFLC2* promoter. *BrFLC2* has been linked to the QTLs for bolting time, budding time, and flowering time [96]. *C. ensifolium* has a highly heterozygous SNP-rich genome. In this study, we identified a total of 16,676 SNPs distributed among 7,519 isotigs. The average distance between SNPs was 8.33 kb. The most common base substitution was C/T (5,136; 30.80%), followed by A/G (4,804; 28.81%), whereas the rarest was T/G (1,575; 9.45%), followed by A/C (1,592; 9.55%) (Table S8). A high frequency of C/T and A/G transitions has also been reported in other plant species [92,97]. A fraction of these sites represent heterozygous alleles, whereas some are possibly mismatches of two members in a multigene family. Therefore, more samples are needed to validate the putative SNP markers.

In conclusion, the sequence collection in the present study is the first major genomic resource for *C. ensifolium* and the largest collection for genus *Cymbidium*. Using Illumina sequencing technology, we surveyed the floral transcriptome of *C. ensifolium*, assembling 101,423 isotigs and annotating 41,873 of these isotigs. These sequences provide a starting point for further investigation of *C. ensifolium* for flowering and floral development. The collection could serve as a foundation for further genomics studies on *C. ensifolium*, and its relatives. Genic-SSRs and genic-SNPs were predicted and their characterizations were analyzed. The 7,936 SSRs and 16,676 putative SNPs predicted in this study provide a solid foundation for molecular marker development in *C. ensifolium*. We believe that this transcriptome dataset will serve as an important public information platform for accelerating research on the gene expression, genomics, and functional genomics of *C. ensifolium*.

## Supporting Information

Table S1 **A total of 9,830 isotigs from *C. ensifolium* transcriptome involved in 126 pathways with reference to *Arabidopsis thaliana*.**
(XLS)

Table S2 **A total of 9,539 isotigs from *C. ensifolium* transcriptome involved in 123 pathways with reference to *Oryza sativa*.**
(XLS)

Table S3 **Representatives putative MADS genes in *C. ensifolium*, including the five classes of genes.**
(XLS)

Table S4 **Representatives of putative flowering-time genes in the *C.ensifolium* transcriptome.**
(XLS)

Table S5 **The functional classification between two groups of orchid isotigs.** One included shared homologs with both P. equestris and C. sinense, and the other presumably was unique to C. ensifolium.
(XLS)

Table S6 **The 12 *C.ensifolium* accessions used in the genic-SSR validation.**
(XLS)

Table S7 **A total of 17 genic-SSRs showing polymorphic among 12 *C.ensifolium* accessions.**
(XLS)

Table S8 **Summary of putative SNP in the *C.ensifolium* transcriptome.**
(XLS)
